# Occupational Therapist Perspectives: Factors Influencing Recovery Following Motor Vehicle Accident Injury

**DOI:** 10.1177/00084174251336049

**Published:** 2025-04-23

**Authors:** Katelyn Bridge, Dorothy Kessler, Tricia Morrison, Michel Lacerte

**Keywords:** Motor vehicles, Noncatastrophic injury, Qualitative research, Recovery, Traffic accidents, Accidents de la route, blessures non catastrophiques, recherche qualitative, récupération, véhicules automobiles

## Abstract

**Background.** Motor vehicle accident (MVA) injuries can result in persistent impairments which contribute to chronic pain, mental health symptoms, and decreased quality of life. Occupational therapists play a key role in the rehabilitation of those injured in MVAs yet there is lack of evidence to inform occupational therapy practice. An explicit understanding of the factors influencing post-MVA recovery from occupational therapists’ perspectives is needed to inform clinical service delivery. **Purpose.** This study addressed the following question: From the perspective of occupational therapists, what factors are identified as influencing recovery following a noncatastrophic injury sustained in an MVA? **Method.** An interpretive descriptive study design was used. Data were collected through semistructured interviews with 10 occupational therapists who provide auto-insurer funded occupational therapy to clients with noncatastrophic injuries from an MVA. Data were analyzed using constant comparative analysis. **Results.** Physical symptoms and accessibility, acceptance, social support, access to occupational therapy, and navigating the insurance system were factors identified as influencing post-MVA recovery. **Conclusion.** This study highlights the importance of using a biopsychosocial lens when working with clients post-MVA. Recovery post-MVA needs to be considered in the context of the insurance system, as navigating the insurance system was a predominant factor influencing recovery.

## Résumé

**Description.** Les blessures causées par un accident de la route peuvent entraîner des déficiences persistantes contribuant à la douleur chronique, à des symptômes psychologiques et à une diminution de la qualité de vie. Les ergothérapeutes jouent un rôle clé dans la réadaptation des personnes blessées dans les accidents de la route, mais il y a peu de données probantes pour éclairer leur pratique dans ce contexte. Une compréhension explicite des facteurs influençant le rétablissement post-accident du point de vue des ergothérapeutes est nécessaire pour éclairer la prestation des services cliniques. **But.** Cette étude porte sur la question suivante : du point de vue des ergothérapeutes, quels sont les facteurs identifiés comme influençant la récupération à la suite d’une blessure non catastrophique découlant d’un accident de la route? **Méthodologie.** Une étude descriptive interprétative a été réalisée. Les données ont été recueillies au moyen d’entrevues semi-structurées avec 10 ergothérapeutes fournissant des services financés par un assureur automobile à une clientèle ayant subi des blessures non catastrophiques dans un accident de la route. Les données ont fait l’objet d’une analyse comparative constante. **Résultats.** Les symptômes physiques et l’accessibilité, l’acceptation, le soutien social, l’accès à l’ergothérapie et la gestion des démarches dans le système d’assurance sont des facteurs qui ont été identifiés comme influençant le rétablissement après un accident de la route. **Conclusion.** Cette étude met en évidence l’importance d’utiliser une optique biopsychosociale dans le travail auprès d’une clientèle ayant subi un accident de la route. La récupération après un accident de la route doit être envisagée dans le contexte du système d’assurance, car les démarches dans le système d’assurance sont un facteur prédominant dans la récupération.

According to Transport Canada (2024), there were an estimated 118,000 injuries resulting from motor vehicle accidents (MVAs) in Canada in the year 2022. Within the province of Ontario alone, an estimated 36,000 individuals were injured in MVAs in the year 2023 (Ministry of Transportation, 2024). Injuries sustained in MVAs can result in persistent impairments which contribute to chronic pain (Giummarra et al., 2020), high prevalance of mental health symptoms (Marasini et al., 2022; Turk et al., 2018), and overall decreased quality of life (Gopinath et al., 2020; Rissanen et al., 2017). Given that MVA-related injuries are a primary cause of disability in Ontario (Ontario Protocol for Traffic Injury Management, 2015), meeting the community-based rehabilitation needs of those injured in MVAs is an important area of development for the occupational therapy profession. A deeper understanding of the factors influencing post-MVA recovery is needed to inform occupational therapy service delivery and is the focus of this study.

Recovery from MVA-related injuries has been characterized as “multi-faceted and includes return to activities of daily living, work and social/leisure activities at preinjury or at a level deemed acceptable by the individual, with minimal ongoing pain and symptoms considering physical and mental health wellbeing” (Smits et al. 2022, p. 3). A broad range of factors have been proposed as impacting injury recovery post-MVA. A systematic review identifying biopsychosocial (BPS) factors associated with poor or nonrecovery after a minor MVA-related injury found that factors demonstrating the strongest associations with poor or nonrecovery were high initial pain intensity, pain duration and severity, preaccident physical and mental health, and pain catastrophizing (Samoborec et al., 2018). A Delphi study involving an expert panel of researchers, health professionals and insurance representatives identified resilience, coping skills, recovery expectations, preaccident health, workplace support, and collaboration between the injured individual, their treating providers and claim handlers as factors relevant to recovery post-MVA (Smits et al., 2022). Financial strain has also been noted in several studies, with those injured facing increased medical expenses and reduced earning capacity due to MVA-related injuries (Hasselberg et al., 2019; Samoborec, Ayton et al., 2019; Samoborec, Winbolt et al., 2019).

Within Ontario, community-based occupational therapy provided to those injured in MVAs is funded privately outside of the public healthcare system by auto-insurers through “accident benefits,” which provide financial coverage for medical and rehabilitation costs, attendant care services, and income replacement post-MVA (Financial Services Regulatory Authority of Ontario, n.d.). In addition to receiving accident benefits, those injured and not at fault can also be simultaneously involved in a tort claim, a lawsuit filed against another party to seek financial compensation for the injuries sustained in the accident.

Several studies have identified recovering within an insurance compensation context (including having an accompanying tort claim) as impacting recovery for those post-MVA. A systematic review exploring the relationship between chronic pain and receiving compensation post-MVA found that involvement in tort claims had the strongest association with higher pain intensity and frequency (Giummarra et al., 2016). Murgatroyd et al. (2015) propose that those with higher self-reported levels of pain or disability experience the insurance claims process as more complicated, difficult to navigate, and are more apt to seek legal advice. Along with perceiving the insurance compensation process as adversarial (Murgatroyd et al., 2011), interactions with insurers may be challenging due to limited communication, wait times for treatment approvals, and high volumes of paperwork, all which contribute to increased stress during post-MVA recovery (Elbers et al., 2015). Insurance-specific factors including funding limitations, lack of understanding the system, and lack of information and guidance have also been identified as challenges to post-MVA recovery in more recent studies (i.e., Lindsay et al., 2016; Samoborec et al., 2019b). Psychological factors associated with the MVA, including anger, blame, and perceived injustice have also been identified as factors negatively impacting health outcomes for those receiving insurance compensation post-MVA (Littleton et al., 2011).

Within existing literature, there is significant heterogeneity related to post-MVA recovery. Use of differing approaches to assess factors impacting recovery and recovery outcomes, as well differing participant populations (i.e., injury types/severity) and insurance schemes (i.e., fault-based vs. no-fault) pose limitations in applying study findings to clinical occupational therapy practice. It remains unclear on what factors clinical practice should focus to promote recovery post-MVA (Smits et al., 2022). Except for the study by Smits et al. (2022) which incorporated occupational therapists as members of their expert panel, there is currently a gap in research related to post-MVA recovery that includes occupational therapists. A more explicit understanding of the factors influencing post-MVA recovery from occupational therapists’ perspective is needed to guide occupational therapy service delivery, clinical decision-making, and selection of the most appropriate therapeutic strategies and approaches. Therefore, this study addressed the following research question: From the perspective of occupational therapists, what factors are identified as influencing recovery following a noncatastrophic injury sustained in an MVA?

This study focused specifically on those with noncatastrophic injuries. The range of injuries deemed noncatastrophic within Ontario's auto-insurance system is broad and includes fractures, concussions, complex soft tissue injuries, chronic pain, and psychological sequelae (i.e., anxiety, depression). Focusing on those with noncatastrophic injuries will allow for the identification of patterns and similarities among a clinical population comprised of a range of injuries, rather than focusing on one specific injury type.

## Method

### Study Design

This study utilized an Interpretive Description (ID) study design. Interpretive Description studies aim to uncover the subjective experience of a population of interest to inform clinical practice (Thompson Burdine et al., 2021). Interpretive Description studies recognize the value of subjective and experiential knowledge as sources of applied practice knowledge (Thorne, 2016). Interpretive Description studies are commonly conducted with small sample sizes, ranging from five to 30 participants (Thorne, 2016). Because the intent is to develop knowledge relevant to answering a practice question, emphasis should be given to who the most appropriate participants are to provide insight into the phenomenon of interest, rather than sample size (Thorne, 2016). Accordingly, a sample size of 10 was established. The biopsychosocial (BPS) model (Engel, 1977) served as a theoretical framework for this study. The Queen's University Health Sciences and Affiliated Teaching Hospitals Research Ethics Board provided ethics approval for this study (approval number REH-870-23).

### Participants

Convenience sampling was used to recruit 10 participants through Ontario-based organizations that provide auto-insurer funded occupational therapy. Organizations were asked to circulate a study flyer to occupational therapy staff who then contacted the primary researcher to express interest. Those who expressed interest were screened by the primary researcher (KB) to ensure eligibility for the study and informed consent was obtained.

To be eligible to participate, occupational therapists were required to work in Ontario and have at least one year of clinical experience providing community-based occupational therapy to clients with noncatastrophic injuries through auto-insurance funding. Those who did not deliver direct occupational therapy treatment, such as those in case management roles, were excluded. Additional inclusion criteria were the ability to participate in a 1-h virtual interview in English.

### Data Collection

Data were collected between July and September 2023 using semistructured interviews of approximately 1 h in length. Interviews were completed by videoconferencing or telephone, based on participant preference. All interviews were conducted by the primary researcher (KB). Interviews were audio-recorded and transcribed by the primary researcher for data analysis. Data collection and analysis were completed concurrently; interview recordings were transcribed and analyzed as the interviews occurred. This allowed for ideas from early interviews to be incorporated into interviews with later participants to gain their perspectives on emerging themes. Given the use of the BPS model, participants were asked about what factors impact the recovery of clients with noncatastrophic injuries, with follow-up prompts inquiring particularly about physical (biological), psychological, and social contextual (personal and environmental) factors.

#### Researcher Positionality

Within ID studies, a researcher's clinical knowledge, research background, and personal experience is considered a source of insight during the research process (Thompson Burdine et al., 2021). The primary researcher is an occupational therapist working in the auto-insurance sector. This may have influenced interactions through easier entrance into the research setting, familiarity with the topic, and a better understanding of reactions of participants (Berger, 2015). To ensure ongoing reflexivity throughout the course of research, use of analytical memos, reflective journaling, and regular consultation with the research team took place to address the synthesis of researcher perspectives and existing knowledge with the data collected from participants.

### Data Analysis

Interview transcripts were uploaded to data analysis software (NVivo 13) which was used to organize and code interview data. Data analysis was completed using constant comparative analysis (Glaser and Strauss, 1967), as ID studies favor the use of an inductive data analysis approach (Thompson Burdine et al., 2021). Constant comparative analysis involves comparing each piece of data (i.e., statement, theme) with all others that may be similar or different to develop relationships among the data. Interview transcripts were reviewed line by line with codes assigned to key ideas. As successive interviews were coded, categories and themes were constructed based on shared or differing ideas between participants, informing the final study results. The first three interviews were coded separately by the primary researcher and secondary researcher (DK) and then discussed together to develop a coding framework and ensure coding consistency. The remaining seven interviews were coded independently by the primary researcher with periodic check-ins with the secondary researcher and review of categories and themes with the research team. Once final categories and themes had been established, they were organized within the three domains of the BPS model.

## Results

Eleven occupational therapists expressed interest in study participation. One was excluded as she worked in a publicly funded setting where occupational therapy was not paid for through auto-insurance funding. Ten occupational therapists were deemed eligible for study participation. All participants identified as female. Ninety percent (*n* = 9) of participants reported providing occupational therapy to clients in a variety of geographic locations (urban, suburban, and rural) while one participant provided services only to clients living in urban settings. Participants had between one and 20 years (mean 7.1 years) of clinical experience working as occupational therapists, and from 1 to 20 years (mean 5.9 years) experience working specifically with clients with noncatastrophic injuries within the auto-insurance sector. Seventy percent (*n* = 7) of participants were within their first five years of clinical practice. Four of 10 participants had worked exclusively in the auto-insurance sector (i.e., had not worked in other practice settings). All participants were employees of a private rehabilitation company. Participants worked out of two cities within Ontario, Canada.

### Factors Influencing Recovery

**Figure 1.**
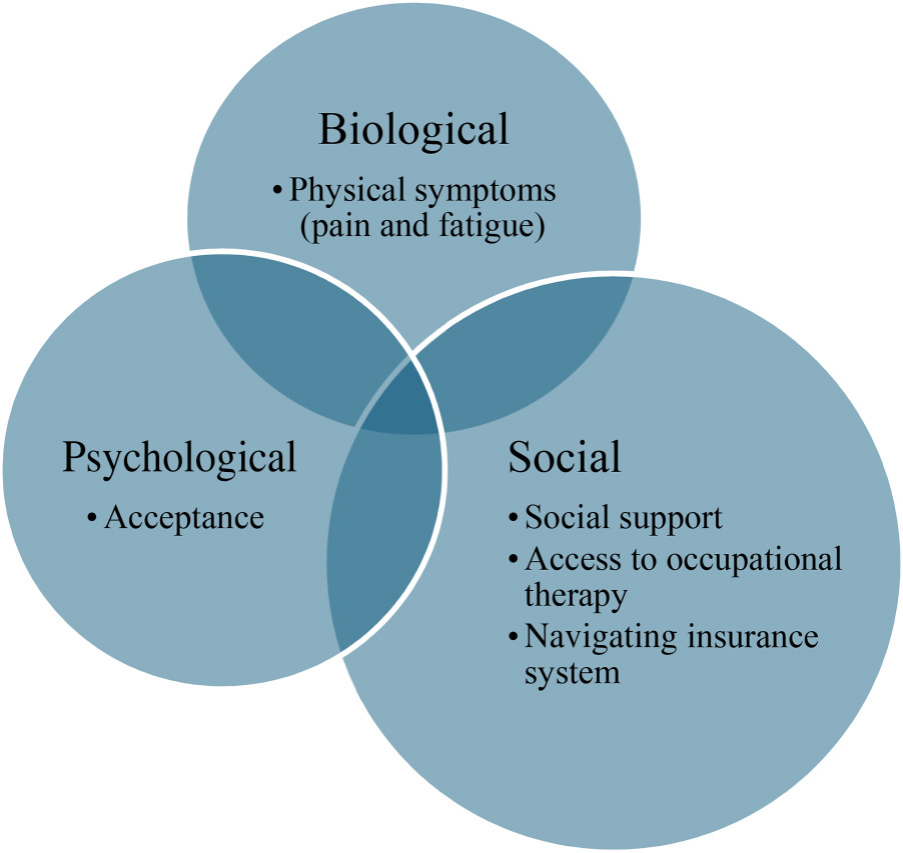
Factors by Biopsychosocial Domain.

The following categories represent factors identified by participants as influencing recovery following a noncatastrophic injury sustained in an MVA: physical symptoms and accessibility, acceptance, social support, access to occupational therapy, and navigating the insurance system. These categories are comprised of themes as summarized in Table 1. While the factors are presented separately, understanding recovery requires consideration of the interaction among the factors, and study findings can be understood using a BPS lens. Figure 1 illustrates how the identified factors fall within the three domains of the BPS model.

**Table 1.** Factors Influencing Recovery and Themes.

Factor	Themes
Physical symptoms and accessibility	Pain and fatigue impact activity participation Lack of accessibility creates recovery barriers
Acceptance	Accepting and making room for symptoms promotes activity engagement Focus on preaccident functioning can impede acceptance
Social support	Social support provides accountability and instrumental support Isolation and not feeling understood make recovery more challenging
Access to occupational therapy	Late referrals lead to poor coping
Navigating the insurance system	The legal voice carries authority Tensions exist between recovery and financial settlement Perceived injustice and distrust can create strained relationships Adjuster support affects access to services

In presenting the study findings, “client(s)” is used to denote recipients of post-MVA occupational therapy. This terminology was selected as participants were specifically describing their experiences in relation to clients to whom they have provided services. Participants were randomly assigned pseudonyms using an online name generator to maintain confidentiality in presenting interview quotes.

### Physical Symptoms and Accessibility

Physical symptoms were described as creating challenges for clients. Two themes were identified: (a) pain and fatigue impact activity participation; and (b) lack of accessibility creates recovery barriers.

*Pain and Fatigue Impact Activity Participation.* Pain and fatigue were described as negatively impacting activity participation. Clients may experience physical restrictions due to their injuries, as noted by Alex: “A lot of clients have chronic pain or broken bones and physically they're not able to take care of themselves.” These symptoms were also cited as negatively impacting motivation to engage in social and community-based activities, resulting in isolation. Sierra reflected, “Avoiding activities leads towards a bit of a downward spiral,” suggesting a connection between pain and fatigue and poorer mental health.

*Lack of Accessibility Creates Recovery Barriers.* When community environments are not physically accessible for clients, this was described as creating barriers to accessing necessary services, including access to healthcare. Alex noted, “One of the biggest barriers would be getting back to driving. If they can’t drive, that creates a really big barrier for them if they don't have access to in-home services.” This creates recovery barriers as in-home services are typically associated with higher rehabilitation costs due to provider travel costs, and therefore may be less likely to be approved by insurers. Physical inaccessibility was suggested as contributing to poorer mental health (specifically depression), as clients have difficulty coping with the fact that “they can't do all the things they physically used to be able to do” (Darcy).

### Acceptance

Acceptance was considered relevant to post-MVA recovery, with two themes identified: (a) accepting and making room for symptoms promotes activity engagement; and (b) focus on preaccident functioning can impede acceptance.

*Accepting and Making Room for Symptoms Promotes Activity Engagement.* Clients accepting changes to themselves (i.e., changed physical abilities) and their life roles postaccident was described as allowing them to be more open to alternative ways of engaging in meaningful preaccident or new activities despite the presence of ongoing symptoms. Sierra noted, “Accepting that they're not the same as they were allows them the space to find alternative activities or alternative ways of doing things.” Accepting and making room for symptoms also involves recognition that recovery is subjective, and that clients “have to decide for themselves what are they going to accept and how are they going to move on” (Alex).

*Focus on Preaccident Functioning Can Impede Acceptance.* A focus on returning to one's preaccident level of functioning was noted as impeding acceptance of limitations. Alex detailed, “They’re not necessarily ready to accept that this is their new reality. That creates a barrier, continuously striving to be the person they were before the accident, which might not be possible.” Striving to be their preaccident selves was viewed as a barrier to the use of therapeutic strategies which promote alternative ways of engaging in preaccident activities, thereby impeding recovery progress. Within occupational therapy treatment, Sierra described “leveling expectations” and cited the need for more negotiation to agree upon realistic goals with these clients.

### Social Support

Two themes were identified related to social support: (a) social support provides accountability and instrumental support; and (b) isolation and not feeling understood makes recovery more challenging.

*Social Support Provides Accountability and Instrumental Support.* Social support was described as providing external accountability and motivation to progress with goals including exercise and participation in community-based activities. Social support was also cited as reducing day-to-day demands to allow a greater focus on recovery. Camilla stated, “When you have social supports, they may be an extra level of accountability to help you with your recovery, but also taking on a load that you don’t have to worry about.” Examples of instrumental support included assistance with transportation, meals, and housekeeping. Clients with increased social supports were perceived as more independent in their ability to access required supports and services. The presence of social support was viewed as positively contributing to recovery, as summarized by Laurel: “It's been my experience that people who have a social network do better, particularly from a mental health standpoint.”

*Isolation and Not Feeling Understood Make Recovery More Challenging.* While social support was viewed to contribute positively to recovery, a sense of isolation, including not feeling understood by their social network, can make recovery more challenging for clients. Rosie described many clients as having delayed progress because they “feel so alone with their symptoms.” For clients with a concussion, their social network may have difficulty understanding their condition which can create mismatched recovery expectations. Family and friends may not understand why a client has not made further recovery progress and may expect them to be fulfilling their preaccident responsibilities. Evelyn stated, “If their significant others think they should just get on with it and they need the help, that can make things really challenging.”

### Access to Occupational Therapy

*Late Referrals Lead to Poor Coping.* A longer period between injury and referral to occupational therapy was described as contributing to poor coping with accident-related injuries. When occupational therapy is initiated after a significant delay, it was perceived that progress may be slower due to the presence of maladaptive coping strategies which must first be addressed. Darcy recounted, “Sometimes it's harder when we get them later on in the recovery to make a positive difference, but also it's sometimes shocking that the strategies we use and teach have never actually been tried years into their recovery.” This suggests that poor coping with accident-related symptoms due to missed occupational therapy involvement may contribute to slowed recovery progress.

Lack of awareness of the role of occupational therapists in supporting clients post-MVA was cited as contributing to late referrals for occupational therapy. Darcy noted, “We need to continue to advocate for our profession because sometimes [occupational therapists] are not the first thing people think of, especially when there's a physical injury.” Phoebe proposed family physicians could have a key role in more regularly referring to occupational therapy post-MVA.

### Navigating the Insurance System

Several aspects of navigating the insurance system were found relevant to post-MVA recovery. The following themes were identified: (a) the legal voice carries authority; (b) tensions exist between recovery and financial settlement; (c) perceived injustice and distrust can create strained relationships; and (d) adjuster support affects access to services.

*The Legal Voice Carries Authority.* Involvement of legal representatives was viewed as necessary to obtain funding approvals when occupational therapists or clients experienced challenges in communication with insurers. It was perceived that legal representatives have authority above that of healthcare professionals, and they are needed to advocate for therapists’ recommendations to obtain insurer approval. Involvement of legal representatives was considered beneficial to clients, as Rosie described: “It's helped a lot of my clients feel more well supported and get more approvals for what they needed.”

*Tensions Exist Between Recovery and Financial Settlement.* Recovery of clients may be influenced by the financial incentives of being injured. Rosie stated, “If there's a lawsuit involved, they may actually be a little bit reluctant to progress too much for fear that it will affect their settlement.” As suggested by Rosie, clients may perceive themselves to be entitled to higher financial settlement if they are more injured, which serves as a deterrent to recovery.

Several participants experienced requests from legal representatives to continue with ongoing occupational therapy despite their recommendation for discharge or to increase therapy frequency to convey increased client needs to the insurer (which could contribute to higher financial settlement). Rosie described:

It's such a fine balance to get [clients] as much better as we can, but at the same time, I've been told many times, “don't let them get too much better” and that's an uncomfortable position because my role is not to mediate their lawsuit, it's to help them progress toward their treatment goals.

These requests create discomfort for participants who seek to balance their clinical recommendations with the requests of legal representatives. Darcy described feeling pressure to “make [legal representatives] happy” to maintain easier working relationships.

A final tension noted was that within the insurance system, clients must “prove” their injuries and disabilities to access treatment funding. This ongoing focus on proving disability to access financial benefits was considered counterproductive to recovery and at odds with occupational therapists’ use of strengths-based treatment approaches.

*Perceived Injustice and Distrust Can Create Strained Relationships.* When insurers decline funding requests for treatment/services, this contributes to clients feeling a sense of perceived injustice, distrust, and lack of support. Perceived injustice was viewed as creating strained client–insurer relationships, with clients feeling that they have limited control over their recovery and viewing the insurer negatively. Evelyn stated, “They might feel like their insurance is intentionally not trying to help them. If they don’t feel supported, they feel like that's hindering their recovery.”

Laurel described negative feelings from clients toward their insurer as “colouring all aspects of their life.” Similarly, Kat suggested that clients who have been involved in MVAs tend to be very claim-focused, which can distract from recovery-oriented goals. The negative feelings of perceived injustice and distrust were ultimately viewed as impeding recovery by impacting the client–insurer relationship and contributing to a preoccupation with negative aspects of their recovery experience.

*Adjuster Support Affects Access to Services.* Clients are assigned a designated insurance adjuster who is responsible for overseeing their claim. There is variability with adjuster support, with some adjusters being viewed as more informed on rehabilitation-related issues and the role of occupational therapy. These adjusters were viewed as more favorably approving access to treatments and services. Other adjusters were viewed as prioritizing cost savings on behalf of the insurer, which often involves declining funding for treatments or services. Darcy illustrated this dichotomy:

Some adjusters are so informed and educated on these rehabilitation issues and they see the client's rehabilitation and recovery as the primary goal. But other times, you can really see where there's a disconnect between what's best for the client's recovery and what's best for the pockets of the insurance company.

Variability among adjusters was also reported with respect to timeliness of approvals and frequency and ease of communication. Adjusters who were difficult to reach or unresponsive were viewed as creating additional barriers for clients to access required services for recovery, often contributing to the need for clients to seek involvement of legal representation.

## Discussion

This is the first known study to explore factors influencing recovery following noncatastrophic injury sustained in an MVA from the perspectives of occupational therapists who provide services to this population through auto-insurance funding. Study findings can be understood using a BPS lens. The sole biological factor identified was the presence of pain and fatigue. Acceptance was identified as a psychological factor influencing recovery. The remainder of the identified factors was social contextual factors, which included social support, access to occupational therapy, and the experience of navigating the insurance system. Study findings underscore that recovering from an MVA cannot be understood in isolation from the context of the insurance system.

Pain and fatigue were cited as creating challenges for activity participation and contributing to accessibility barriers which can further contribute to poor mental health. High pain intensity, duration and severity, and pain catastrophizing have been identified as contributing to poor recovery following minor injury in an MVA (Samoborec et al., 2018) and a link has also been proposed between involvement in a tort claim post-MVA and increased levels of pain (Giummarra et al., 2016). The presence of pain in clients post-MVA should be addressed as research suggests that those with prolonged recovery and/or higher pain were more likely to find the insurance process complicated and difficult to navigate (Murgatroyd et al., 2015).

A study exploring the meaning of acceptance for individuals with chronic pain found that acceptance can have several meaning structures, including as a personal empowerment process, but also as a threat and personal failure (Biguet et al., 2016). These conceptualizations of acceptance reflect the dichotomy described in this study where acceptance of symptoms was perceived as allowing clients to move forward despite symptoms, while clients who were focused on their preaccident status were considered to have low levels of acceptance which created barriers to therapeutic progress. This latter group may perceive acceptance of their injuries/symptoms as unacceptable and incongruent with their recovery expectations (Biguet et al., 2016). Prior research within the auto-insurance context has identified similar concepts to acceptance including coping style, resilience, and recovery expectations as relevant to post-MVA recovery (Smits et al., 2022).

The findings that social support is beneficial during post-MVA recovery are congruent with existing studies (Alharbi et al., 2019; Brown et al., 2020; Murgatroyd, Cameron et al., 2011; Murgatroyd, Lockwood et al., 2015). Social support post-MVA has been found to be associated with better physical health outcomes (Prang et al., 2015) and reduced allied healthcare service use (Prang et al., 2016). This is akin to the perception of clients as requiring less therapist support when social support was available identified in this study. Both the benefits of social support and the challenges with not feeling understood by one's support network were identified, suggesting that it is not merely the presence of social support but is instead the characteristics of that social support, which serves as a positive influence on recovery.

Legal representatives were recognized as carrying authority within the auto-insurance context, supporting clients to access increased financial benefits. The perceived necessity of legal representation in navigating insurance processes has been identified in several studies (Murgatroyd, Cameron et al., 2011; Murgatroyd, Lockwood et al., 2015). Financial settlement was viewed as contributing to reluctance to recover. Previous research suggests that participation in an insurance claim and consulting a legal representative may create disincentives for recovery and contribute to increased psychological stress (Littleton et al., 2011). Needing to prove injuries and disability for the purposes of accessing accident benefits may inadvertently perpetuate poor recovery, as this requires clients to focus on the discrepancies between their former and current selves (which was identified as impeding recovery progress) and may further hinder acceptance. Notably, there were also tensions described whereby legal representatives at times attempted to influence the therapeutic process to facilitate a larger financial settlement for clients. This tension may be compounded given that legal representatives are common referral sources for post-MVA occupational therapy. Occupational therapists need to be aware of these influences and ensure that their primary obligation remains to their clients (College of Occupational Therapists of Ontario, 2023).

Insurance adjuster support was identified as relevant to recovery and was primarily appraised in terms of their willingness to approve funding requests for recommended services/treatment. Lack of communication, lack of information about processes, and long wait times for approvals results in dissatisfaction with the insurance process (Elbers et al., 2015). Participants in this study expressed an expectation that their requests *should* be approved and if they are not, this indicates lack of insurance adjuster support. Given that perceived injustice and distrust were highlighted as potential barriers to the client–insurer relationship, occupational therapists must ensure that they are not inadvertently perpetuating negative feelings. Occupational therapists need to ensure that their own relationships or perceptions of insurers and legal representatives do not impact clients’ relationships.

This study aimed to identify factors influencing recovery to inform clinical decision-making for occupational therapists. Findings highlights the complexity of recovery within the insurance context post-MVA. For occupational therapists challenged by clients who are demonstrating poor injury recovery, these findings may provide insight into influences outside of the direct client–therapist relationship which impact recovery. Occupational therapists should reflect critically on how the identified factors arise and interact in their work with their post-MVA clients, and on which factors they can most directly intervene. Given the importance of social support, occupational therapists should consider how they can enable clients to access existing supports or building new support networks. Similarly, occupational therapists can employ mental-health-based strategies, such as those deriving from acceptance and commitment therapy (ACT) to promote acceptance of post-MVA limitations and proactively address feelings of perceived injustice which may otherwise impede recovery progress. While ACT has not been researched specifically in the post-MVA context, a core tenet of ACT is to focus on improved functionality rather than symptom reduction (Kangas & McDonald, 2011), which aligns with the concept of “making room” for symptoms as part of acceptance, as described in this study. Previous research suggests that stress and poor recovery outcomes could be mitigated by more proactive claims management approaches to improve communication between clients and insurers and decrease the perceived adversarial nature of recovery within the insurance context (Elbers et al., 2015; Murgatroyd et al., 2011; Samoborec et al., 2019b; Sim et al., 2024). Occupational therapists interact with all parties within the insurance context including clients, legal representatives, and insurers and are uniquely positioned to support relationship management between clients, their legal representatives, and insurers. Perceived injustice was cited when clients felt they were not receiving what they needed or experienced delayed access to care. Reviewing with clients the existing insurance regulations (i.e., accident benefits coverage, approval timelines) may serve to proactively reduce frustrations. Occupational therapists should self-reflect and consider treatment recommendations carefully to avoid further contributing to the insurance denials that lead to clients feeling poorly supported. Occupational therapists can also continue to advocate for the role of occupational therapy post-MVA to decrease instances of late referrals which can contribute to poor coping and ultimately more complex recovery. Clients may also benefit from education on the factors identified to enhance their understanding of influences at play in the recovery process.

### Limitations

This study had several limitations. This study is limited to a small number of occupational therapists in Ontario, Canada, and findings may not reflect broader experiences, particularly, those practicing in different geographical and insurance contexts. Participants were from the same organization (across two geographical sites), meaning participants will have had the same clinical leadership and direction for therapy. Due to varying organizational factors, occupational therapists from other organizations may have differing perspectives not captured in this study. This study does not incorporate the perspectives of insurers who may offer additional insights into the factors influencing recovery related to navigating the insurance system. Recovery experiences of those who do not participate in occupational therapy post-MVA may also differ from those explored in this study. To complement this study, further research is recommended to incorporate the injured client perspective on factors influencing recovery. A separate component of this study examines this and will be reported elsewhere.

## Conclusion

The findings of this study highlight the importance of a BPS lens when working with clients following noncatastrophic injuries sustained in an MVA. Factors influencing recovery were identified within the biological, psychological, and social contextual domains. Recovering from an MVA cannot be understood in isolation from the context of the insurance system, as navigating the insurance system was found to be a predominant factor influencing recovery. The authority of legal representatives, tensions between financial settlement and recovery, perceived injustice, and the supportiveness of insurers was insurance-specific themes found to impact recovery. Occupational therapists should reflect critically on how the factors identified in this study present in their clinical work with clients post-MVA, and on which factors they can most directly intervene. Recognition of the influences of contextual factors will support occupational therapists with clinical decision-making and provides additional insight into the recovery experience of clients post-MVA.

## Key messages

- Factors influencing post-MVA recovery were identified within the biological, psychological, and social contextual domains. Understanding recovery requires consideration of the interaction among these factors.
- Occupational therapists should reflect critically on how the identified factors arise and interact in their work with post-MVA clients and on which factors they can most directly intervene. Recommendations include encouraging use of social support, promoting acceptance, mitigating perceived injustice, and supporting relationship management between clients, insurers, and legal representatives.
